# Advanced Planar Projection Contour (PPC): A Novel Algorithm for Local Feature Description in Point Clouds

**DOI:** 10.3390/jimaging10040084

**Published:** 2024-03-29

**Authors:** Wenbin Tang, Yinghao Lv, Yongdang Chen, Linqing Zheng, Runxiao Wang

**Affiliations:** 1 School of Mechanical and Electrical Engineering, Xi’an Polytechnic University, Xi’an 710043, China; 210211023@stu.xpu.edu.cn (Y.L.); chenyd@xpu.edu.cn (Y.C.); 210221038@stu.xpu.edu.cn (L.Z.); 2 School of Mechanical Engineering, Northwestern Polytechnical University, Xi’an 710060, China; wangrx_nwpu@126.com

**Keywords:** local reference frame, local feature descriptor, convex hull contour

## Abstract

Local feature description of point clouds is essential in 3D computer vision. However, many local feature descriptors for point clouds struggle with inadequate robustness, excessive dimensionality, and poor computational efficiency. To address these issues, we propose a novel descriptor based on Planar Projection Contours, characterized by convex packet contour information. We construct the Local Reference Frame (LRF) through covariance analysis of the query point and its neighboring points. Neighboring points are projected onto three orthogonal planes defined by the LRF. These projection points on the planes are fitted into convex hull contours and encoded as local features. These planar features are then concatenated to create the Planar Projection Contour (PPC) descriptor. We evaluated the performance of the PPC descriptor against classical descriptors using the B3R, UWAOR, and Kinect datasets. Experimental results demonstrate that the PPC descriptor achieves an accuracy exceeding 80% across all recall levels, even under high-noise and point density variation conditions, underscoring its effectiveness and robustness.

## 1. Introduction

Point cloud local feature descriptors are pivotal in the computer vision field [1]. They are derived by converting local geometric information into attributes such as curvature [2], eigenvalue [3], and the density of spatial points [4]. Unlike global feature descriptors, local descriptors offer enhanced robustness against occlusions and missing local information in models. Owing to these benefits, local feature descriptors have found extensive applications in object detection [5], point cloud registration [6,7], and cultural heritage restoration [8].

Recent research by both domestic and scholars has significantly advanced the study of point cloud local feature descriptors, broadly categorizing them into two types [9]. The first type encompasses descriptors independent of the Local Reference Frame (LRF), which quantify local geometric features statistically. However, this approach often overlooks the spatial location of point clouds, leading to issues like inadequate description or reduced robustness against rotation and translation. Examples of such descriptors are the spin image (SI) [10], Fast Point Feature Histograms (FPFH) [11], and 3D Shape Context (3DSC) [12].

The second category includes descriptors that rely on an LRF, transforming neighboring points’ information into a consistent LRF to achieve spatial position invariance. This method encodes the local attributes of neighboring points to represent the query point’s local feature. Descriptors in this category, such as the Signature of Histograms of Orientations (SHOT) [13], Geometric Feature Statistics Histogram (GFSH) [14], and Weighted Height Image (WHI) [15], leverage the LRF to fully decode spatial position information, offering advantages like invariance to rigid transformations, improved stability, and enhanced expressiveness. Despite these advancements, challenges remain, including limited robustness to noise and point density variations, extensive descriptor dimensions, and decreased computational efficiency.

Motivated by the aforementioned challenges, this study introduces a novel method named Planar Projection Contour (PPC), distinguished by its enhanced descriptiveness and resilience to issues like noise and variations in point density. The PPC approach begins with the construction of an LRF through the analysis of local neighboring points around a key point. Subsequently, these neighboring points are projected onto three orthogonal planes defined by the LRF. The two-dimensional projections on each plane are then modeled into convex hull contours. By concatenating these three convex hull contours, a comprehensive feature vector is formed, resulting in the generation of the PPC descriptor. The PPC’s effectiveness is rigorously evaluated against traditional descriptors across three public datasets, with the experimental outcomes indicating superior performance in tasks such as shape retrieval and alignment. This study introduces a novel local feature descriptor named PPC, designed to offer a harmonious blend of descriptiveness, robustness, and computational efficiency. Additionally, we present a comparison method specifically tailored for evaluating feature similarity based on convex hull contours, applicable to descriptors that utilize these contours as features.

The structure of this paper is organized as follows: Section 2 provides an overview of existing 3D local feature descriptors. Section 3 details the methodology behind the proposed PPC descriptor. Section 4 discusses the experimental evaluation of PPC in comparison to traditional descriptors across three public datasets. Finally, conclusions are presented in Section 5.

## 2. Related Work

A substantial body of research focuses on the comprehensive analysis of point cloud local feature descriptors, with each descriptor offering a distinct representation of local information. Based on the coding space of their attributes, current descriptors are categorized into two types: those operating in the 3D space domain and those in the 2D space domain.

Three-dimensional space domain descriptors characteristically partition the local vicinity of neighboring points into multiple subspaces using varied approaches to separately encode and, thus, represent features. Salti S et al. [13] introduced the SHOT descriptor, which employs a spherical bounding box to segment the local space around a key point into 32 subspaces based on radial, azimuth, and elevation directions. It then quantifies the cosine similarities between the neighboring points and the key point in each subspace. Building on the SHOT framework, the B-SHOT descriptor was developed, utilizing a method of byte-by-byte comparison for enhancement. B-SHOT [16] was generated based on the SHOT descriptor using the method of comparing individual byte sizes one by one. Zhao H et al. [17] proposed the HoPPF descriptor, segmenting the local point pairs associated with each key point into eight regions and crafting sub-features based on the distribution of these pairs within each zone. While SHOT, B-SHOT, and HoPPF leverage histogram features, their discriminatory power is somewhat curtailed by the mutual interference of adjacent histograms. Tang K et al. [18] introduced the SGC descriptor, which constructs a cube around the key point’s local support surface, subdividing it into evenly distributed grids to count geometric centroid values, vertex coordinates, and vertex numbers. Quan S et al. [19] put forward the LOVS descriptor, creating a uniformly gridded cube from the key point’s local surface and dividing it into a set of uniformly distributed voxels. Both SGC and LOVS approach the local surface construction of point clouds through cubic segmentation, which often leads to largely unoccupied meshes in edge areas, reflecting a lower spatial utilization.

Two-dimensional space domain descriptors transform 3D shapes into 2D space through projection, simplifying the 3D coordinate information of a point cloud into features like images and contours, which are then encoded. Johnson A E et al. [10] introduced the spin image (SI) descriptors, which create a 2D grayscale map by projecting neighboring points onto horizontal and vertical 2D planes using the normal vector of the key point as a reference axis. Guo Y et al. [20] developed the Rotational Projection Statistics (ROPS) descriptor, which involves continuously rotating the local surface within the LRF and projecting the surface after each rotation to calculate point density statistics. Additionally, Guo Y et al. [21] proposed the triple spin images (TriSI) descriptor, achieving a comprehensive description by generating three spin images around different axes. Yang J et al. [22] introduced the Triple Orthogonal Local Depth Images (TOLDI) descriptor, which calculates the LRF for the point cloud’s local surface using normal vectors and weighted projection vectors, effectively decoding rotation invariance and spatial information. Further, Yang J et al. [23] proposed the Rotational Contour Signatures (RCS) descriptor, characterizing the 3D shape through multi-view information obtained via rotation and by counting the 2D contours at each viewpoint. The method of mapping 3D local coordinate information into 2D simplifies descriptor computational complexity and accelerates encoding. However, this approach may result in significant loss of local information, leading to reduced robustness against noise, point density variations, and other factors.

In light of the analyzed strengths and weaknesses of 2D and 3D space domain descriptors, this work proposes the PPC descriptor, which enhances robustness by encoding convex hull contours of 2D projection points from three orthogonal views. This strategy not only preserves high descriptiveness but also simplifies the encoding of point cloud local information, addressing the common robustness issues found in many 2D space domain descriptors.

## 3. Construction of PPC

### 3.1. Overall Construction Process

**Figure 1:**
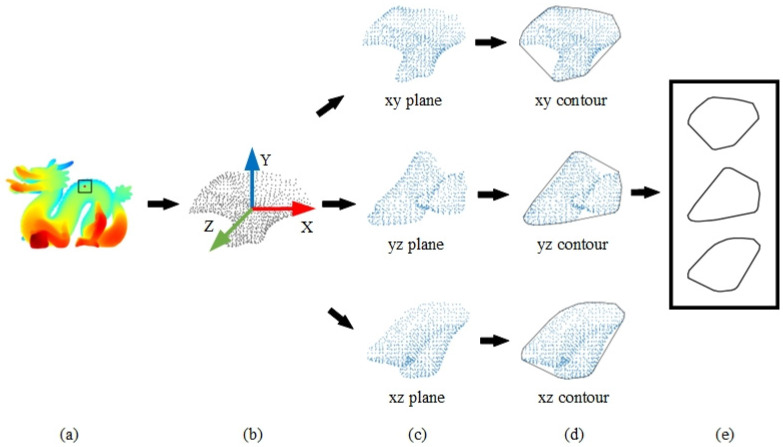
PPC descriptor construction flowchart.((**a**), Object and key point. (**b**), LRF was constructed based on key Point and neighboring points. (**c**), Projection points of neighboring points onto three planes. (**d**), Contours of neighboring points. (**e**), Concatenation of convex hull contours).

The process of constructing the PPC descriptor unfolded in several key steps. Initially, the LRF was determined by analyzing the covariance between the query point and its neighboring points. Subsequent to this, the neighboring points were projected onto the LRF’s three orthogonal planes. The culmination of this process involved fitting the 2D projection points on each plane into convex hull contours. The PPC descriptor was then formulated through the systematic concatenation of these contours. A visual representation of the PPC construction workflow is provided in Figure 1.

### 3.2. Construction of LRF

The LRF played a crucial role in embedding spatial position information into the local feature representation of a point cloud. This spatial information, once anchored to the LRF, became invariant to rotation and translation, significantly enhancing the descriptor’s robustness and utility. The method used to establish the LRF included precise calculations for the X-axis, Y-axis, and Z-axis components.

The Z-axis was computed as the normal vector of key point *P*, which could be obtained via covariance on the neighboring points *P_i_* of *P* [13].

To determine the the X-axis, the neighboring points *P_i_* were projected on the tangent plane defined by the Z-axis. For each projected point, a projection vector *V_i_* was computed, which is shown in Formula (1).

(1) $$V_{i}=PP_{i}-(PP_{i}\cdot Z)\cdot Z$$

where *P* denotes the key point, and *P_i_* denote neighboring points. Z denotes the Z-axis vector.

We assigned weights $W_{i}^{1}$ according to the Euclidean distances from *P_i_* to *P*. We also assigned weights $W_{i}^{2}$ based on the projection distances from *P_i_* to the tangent plane of the Z-axis. Then, the weighted projection vectors were summed and normalized to obtain the X-axis, which is shown in Formulas (2) and (3).

(2) $$X=\sum_{i=1}^{K}W_{i}^{1}W_{i}^{2}V_{i}/\left\|\sum_{i=1}^{K}W_{i}^{1}W_{i}^{2}V_{i}\right\|$$

(3) $$\left\{\begin{array}{l} W_{i}^{1}={\left(r-\left\|p_{i}-p\right\|\right)}^{2}\\ W_{i}^{2}={\left(PP_{i}\cdot Z\right)}^{2} \end{array}\right.$$

where *r* denotes search radius of the key point. *K* denotes the number of neighboring points.

The Y-axis was determined by calculating the cross-product between the Z-axis and the X-axis. This method ensured that the Y-axis was orthogonal to both the Z-axis and X-axis, thereby completing the orthogonal basis of the LRF and establishing a robust coordinate system for representing the point cloud’s local geometric features.

### 3.3. Construction of PPC

**Figure 2:**
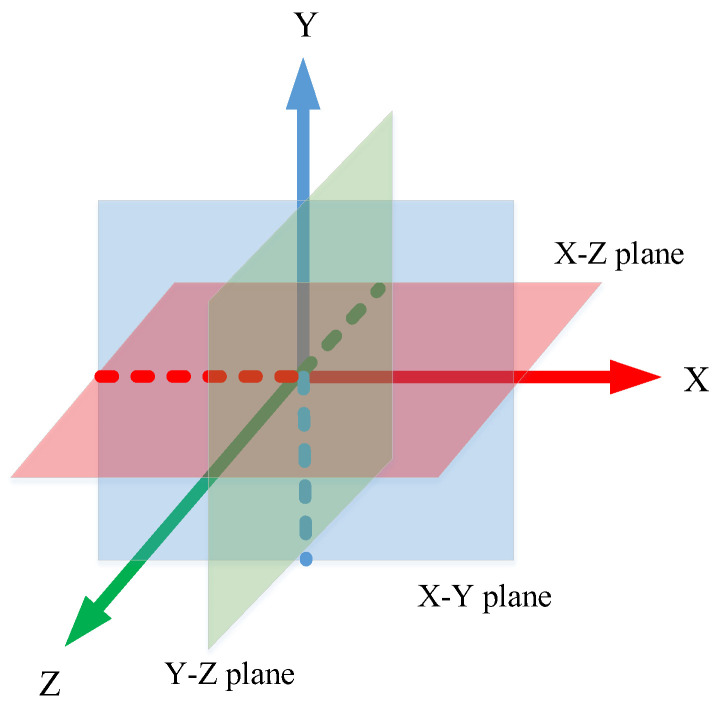
Orthogonal views of three planes. (Light red, light blue, and light green planes represent the X-Z, X-Z, and Y-Z planes, respectively).

In Section 3.1, the construction of the LRF segregated the local support plane, created by the key point and its neighboring points, into three orthogonal planes: the X-Y plane, the Y-Z plane, and the X-Z plane. This division facilitated a comprehensive analysis of the point cloud’s local geometry from multiple perspectives, as depicted in Figure 2.

The projection points on the three planes were obtained by projecting the neighboring points *P_i_* onto the X-Y plane, Y-Z plane, and X-Z plane, respectively. This process was mathematically represented in Formula (4), which provided a systematic approach for capturing the local geometric characteristics of the point cloud from different orientations, ensuring a thorough and multidimensional feature extraction.

(4) $$\left\{\begin{array}{l} J_{xy}=\left\{(x,y)|(x,y,z)\in P_{i}\right\}\\ J_{yz}=\left\{(y,z)|(x,y,z)\in P_{i}\right\}\\ J_{xz}=\left\{(x,z)|(x,y,z)\in P_{i}\right\} \end{array}\right.$$

where *J_xy_*, *J_yz_*, and *J_xz_* denote the set of projected points of *P_i_* in the X-Y plane, Y-Z plane, and X-Z plane, respectively.

The sets *J_xy_*, *J_yz_*, and *J_xz_* contained a number of points equal to the original count of neighboring points, resulting from the transformation of 3D coordinate information into 2D information through projection. This transformation, while simplifying the data, did not remove the inherent vulnerabilities to noise, variations in point density, missing information, and other perturbations common to both 3D and 2D coordinate systems. To mitigate these vulnerabilities, this study employed the convex hull algorithm to transform the projected 2D point sets *J_xy_*, *J_yz_*, and *J_xz_* into convex hull contours [24], as elaborated in Formula (5). This approach effectively summarized the spatial distribution of points while enhancing the robustness of the descriptor against the aforementioned factors.

(5) $$\left\{\begin{array}{l} C_{xy}=CONVEX\{p_{1},p_{2},\cdots p_{n}|p\in J_{xy}\}\\ C_{yz}=CONVEX\{p_{1},p_{2},\cdots p_{n}|p\in J_{yz}\}\\ C_{xz}=CONVEX\{p_{1},p_{2},\cdots p_{n}|p\in J_{xz}\} \end{array}\right.$$

where *C_xy_*, *C_yz_*, and *C_xz_* denote the convex hull contour of *J_xy_*, *J_yz_*, and *J_xz_*, respectively. *P*_1_, *P*_2_, *… P_n_* denote the edge points of the set of projected points. *CONVEX* denotes the convex hull algorithm.

**Figure 3:**
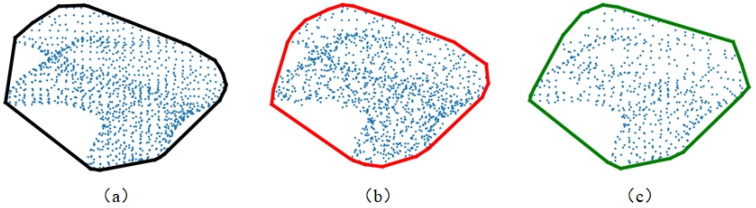
Convex hull contour comparison schematic.((**a**), Original convex hull contour. (**b**), Convex hull contour after adding Gaussian noise. (**c**), Convex hull contour after adding down sampling).

The adoption of convex hull contours for representing the 2D projected point set offers significant benefits, which are outlined as follows:

(1) Utilizing convex hull contours allowed for the internal characteristics of the point set to be disregarded, substantially simplifying the representation of the 2D point set’s geometric information. This simplification led to an improvement in computational efficiency by focusing on the external boundary of the point distribution, as demonstrated in Figure 3a.
(2) Convex hull contours exhibited increased stability when faced with noise interference, in contrast to raw coordinate information. By encapsulating the outermost points, the convex hull effectively minimized the impact of outliers or noise within the data, ensuring a more consistent representation, as illustrated in Figure 3b.
(3) The representation via convex hull contours proved to be more resilient to variations in point density. Unlike methods that rely on the detailed arrangement of points, the convex hull approach maintained a consistent outline, regardless of the density of points within the contour. This robustness was critical for ensuring reliable feature extraction across datasets with varying point densities, as shown in Figure 3c.

Finally, the *C_xy_*, *C_yz_*, and *C_xz_* of the three planes were encoded as the local features of *P*, forming the PPC descriptor.

(6) $$D=\left\{C_{xy},C_{yz},C_{xz}\right\}$$

### 3.4. Feature Matching of PPC

Ideally, the local features of corresponding points between the model point cloud and the scene point cloud should match perfectly. However, in practice, differences in the local features of these corresponding points frequently arose due to factors such as noise interference and missing local information. These discrepancies can challenge the process of accurately matching and recognizing patterns within and between point clouds, underscoring the importance of developing robust feature descriptors capable of mitigating the effects of such imperfections. Hence, it was essential to obtain the optimal feature matching degree by utilizing descriptors to extract the local features of the point cloud, thereby establishing pairs of points based on feature matching. This approach aimed to maximize the similarity between corresponding features, even in the presence of noise and incomplete data, facilitating accurate point cloud registration and recognition tasks. Given a certain pair of feature points *T_i_* and *T_j_*, the local features *D_i_* and *D_j_* were obtained based on PPC. Then, the matching degree of the convex hull contour of both corresponding to the three planes was obtained by accumulating the matching degree of the convex hull contour of the corresponding three planes. The details are shown in Formulas (7) and (8).

(7) $$\left\{\begin{array}{l} M_{1}=S_{xy}^{\Delta }/(S_{xy}^{i}+S_{xy}^{j}-S_{xy}^{\Delta })\\ M_{2}=S_{yz}^{\Delta }/(S_{yz}^{i}+S_{yz}^{j}-S_{yz}^{\Delta })\\ M_{3}=S_{xz}^{\Delta }/(S_{xz}^{i}+S_{xz}^{j}-S_{xz}^{\Delta }) \end{array}\right.$$

(8) $$M(D_{i},D_{j})=\sum_{i=1}^{3}M_{i}$$

where *M*_1_, *M*_2_, and *M*_3_ denote the feature matching values corresponding to the convex hull contours, respectively. $S_{xy}^{\Delta }$, $S_{yz}^{\Delta }$, and $S_{xz}^{\Delta }$ denote the overlap area of the convex hull contours. $S_{xy}^{i}$, $S_{yz}^{i}$, and $S_{xz}^{i}$ are the areas of the X-Y plane, Y-Z plane, and X-Z plane convex hull contours of the feature point *T_i_*. $S_{xy}^{j}$, $S_{yz}^{j}$, and $S_{xz}^{j}$ are the areas of convex hull contours of the X-Y plane, Y-Z plane, and X-Z plane of the feature point *T_j_*. $M(D_{i},D_{j})$ denotes the total feature matching of descriptor *D_i_* and *D_j_*.

**Figure 4:**
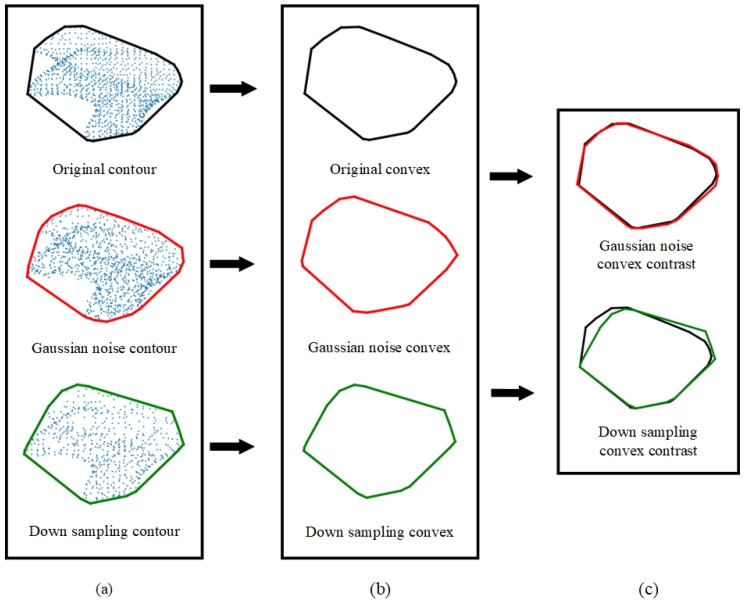
Add noise or downsampled convex matching.((**a**,**b**) The contours and convex hulls under original condition, with added Gaussian noise, and after down sampling. (**c**), Comparison of the overlapping areas between the original convex hull contours and the convex hull contours after adding Gaussian noise, as well as after down sampling).

To demonstrate the resilience of convex hull contour features against noise and variations in point density, an experiment was conducted where a pair of feature points—one from the model point cloud and one from the scene point cloud—was selected. The experiment tested the matching degree of planar convex hull contours subjected to Gaussian noise and downsampling, respectively. This procedure aimed to highlight the robustness of these features under conditions that typically challenge the integrity of point cloud data. The results of this testing are illustrated in Figure 4, providing visual evidence of the effectiveness of convex hull contours in maintaining reliable feature matching in less-than-ideal data quality scenarios.

## 4. Performance Testing of PPC

In this section, the effectiveness of the PPC descriptor is evaluated through a series of tests. Initially, the dataset and evaluation criteria for the experiment are carefully selected. Following this, an analysis is conducted of how key parameters of the PPC influence the descriptor’s performance, utilizing the established criteria. Subsequently, the resilience of PPC and several traditional descriptors, namely TOLDI [20], LOVS [23], SHOT [13], B-SHOT [16], and SGC [22], to noise and variations in point density is assessed using the chosen dataset. Additionally, the computational efficiency of these descriptors is examined. All experiments conducted in this study leverage the capabilities of the open3d [25] point cloud data processing library, ensuring a standardized and reproducible framework for evaluation.

### 4.1. Datasets and Standards

**Figure 5:**
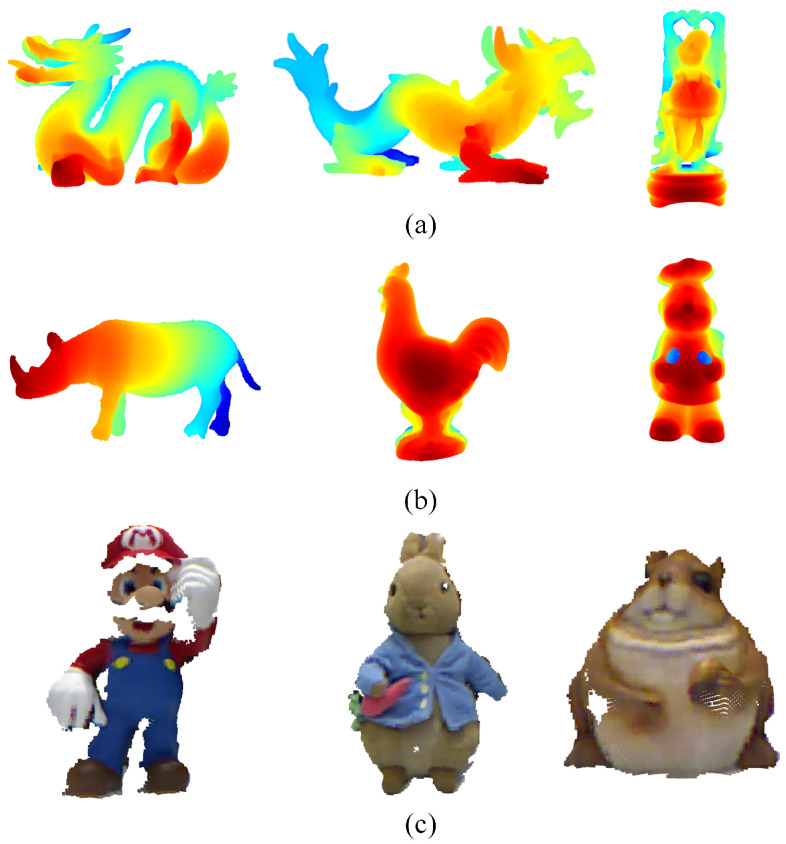
Example of point cloud dataset.((**a**), B3R dataset. (**b**), UWA dataset. (**c**), Kinect dataset).

The datasets used in this experiment include the B3R dataset [25], the UWAOR dataset [26], and the Kinect dataset [27]. A partial dataset is shown in Figure 5.

The RP curve, a recognized benchmark in descriptor performance analysis, offers insights into the trade-off between recall (the ability to retrieve relevant instances) and precision (the accuracy of the retrieved instances). The methodology for calculating the RP curve is detailed below, following the protocols outlined in prior studies [17].

The process begins with the model point cloud, the scene point cloud, and the transformation matrix that defines the relationship between them. From the model point cloud, 1000 points are randomly selected as model key points. For each model key point, the point within the scene point cloud that is closest in terms of Euclidean distance is identified as its corresponding scene key point, forming a pair of key points. The PPC features for each key point pair are then extracted and compared. The model key point features are matched against all features in the scene point cloud to find the nearest and the second nearest feature matches based on feature similarity.

A threshold is applied to the ratio of the distance to the nearest feature vs. the distance to the next nearest feature. If this ratio falls below the set threshold, the model and scene key points are considered a matching pair *M*. A correct match *TM* is identified if the Euclidean distance between the matched points is below a certain criterion, indicating a successful match; otherwise, it is classified as a false match *FM*. By varying the threshold, a series of data points is generated, from which the RP curve is constructed.

Recall is defined as the proportion of true positive matches out of all positive instances in the data, while 1 − Precision reflects the rate of false positive matches in relation to all retrieved instances. Mathematically, these metrics provide a comprehensive overview of a descriptor’s performance across different levels of stringency in feature matching, highlighting the PPC’s ability to identify and correctly match point cloud features under various conditions.

(9) $$Recall=\frac{length(TM)}{length(Pair)}$$

(10) $$1-Precision=\frac{length(FM)}{length(M)}$$

**Figure 6:**
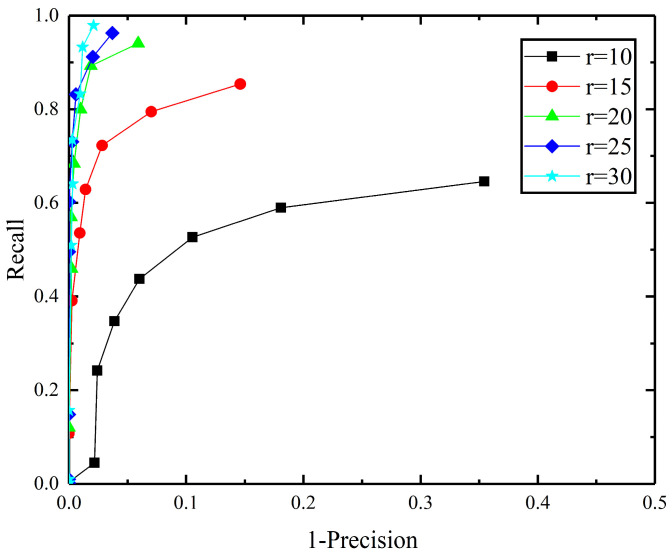
Support radius *r* parameter selection.

The support radius *r* is a key parameter involved in defining the performance of the PPC, which determines the information of the neighborhood points, thus affecting the convex hull contours. In the following, *r* is analyzed in detail. Based on the B3R dataset, after adding the 0.1 mr (mr denotes mesh resolution) Gaussian noise to the scene point cloud and 1/2 downsampling, the performances of the PPC with *r* = 10 mr, 15 mr, 20 mr, 25 mr, and 30 mr are tested, respectively. The results are shown in Figure 6.

Figure 6 reveals that the performance of the Planar Projection Contour (PPC) descriptor is suboptimal at support radii of *r* = 10 mr and *r* = 15 mr. This observation suggests that smaller values of *r* result in fewer neighboring points being included within the descriptor’s calculation range. Consequently, this scarcity of neighborhood points leads to diminished robustness in the convex hull contours derived from these points. As the support radius *r* increases, the descriptor incorporates a larger pool of neighboring points, enhancing the stability of the convex hull contours. This expansion directly contributes to improved feature matching capabilities of the PPC descriptor. Considering this analysis, a support radius of *r* = 10 mr is identified as the optimal setting for constructing the PPC, striking a balance between the inclusivity of neighborhood information and computational efficiency.

In Section 4.2, Section 4.3 and Section 4.4 comparative tests are conducted between PPC and other established descriptors, including SGC, LOVS, SHOT, B-SHOT, and TOLDI. These comparisons aim to highlight the distinctive advantages and performance characteristics of PPC in relation to its peers. The specific parameters utilized for each descriptor in the comparative analysis are detailed in Table 1, providing a clear framework for understanding the experimental setup and the basis for performance evaluation.

### 4.2. Robustness Testing against Gaussian Noise

**Figure 7:**
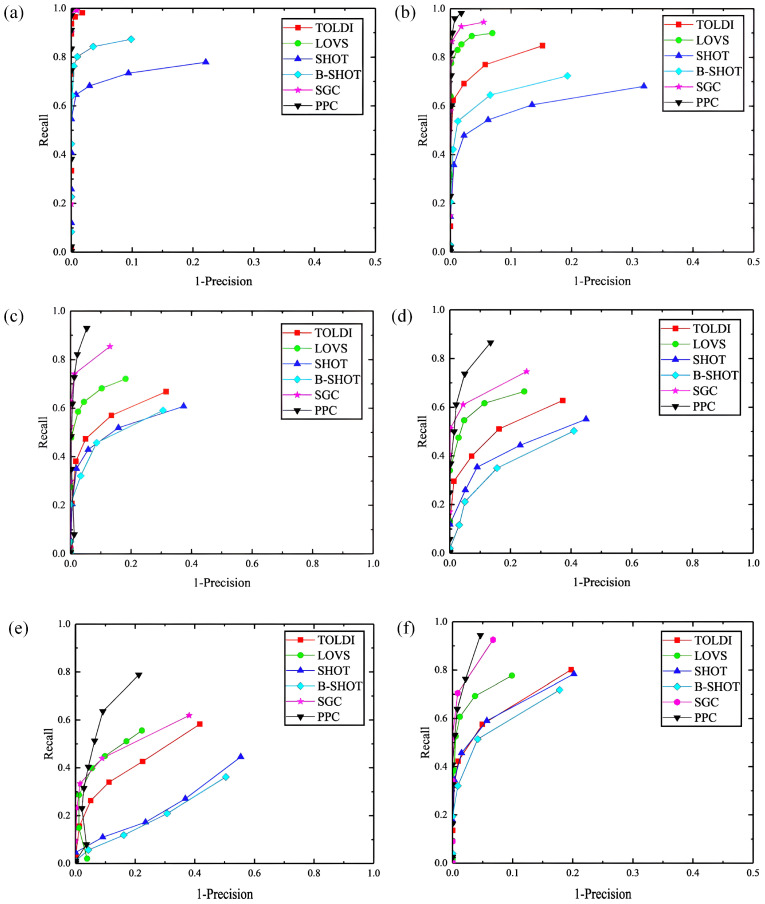
Robustness testing against Gaussian noise. ((**a**),B3R dataset without Gaussian noise. (**b**), B3R dataset with 0.1 mr Gaussian noise. (**c**), B3R dataset with 0.3 mr Gaussian noise. (**d**), B3R dataset with 0.5 mr Gaussian noise. (**e**), UWA dataset with 0.3 mr Gaussian noise. (**f**), Kinect dataset with 0.3 mr Gaussian noise).

To assess the Planar Projection Contour (PPC) descriptor’s resilience against Gaussian noise, this study utilizes the B3R dataset to conduct feature matching performance tests on the scene point cloud under various conditions: without noise and with Gaussian noise with standard deviations of 0.1 mr, 0.3 mr, and 0.5 mr. These results are then benchmarked against established descriptors such as SGC [18], LOVS [19], SHOT [13], B-SHOT 16, and TOLDI [22]. The corresponding Recall vs. 1 − Precision (RP) curves generated from these experiments are displayed in Figure 7a–d. Further, the robustness of PPC to Gaussian noise is examined using the UWAOR and Kinect datasets, with a Gaussian noise of 0.3 mr applied to the scene point clouds.

Analysis of Figure 7a–d reveals that under a low-noise scenario (0.1 mr), descriptors such as SGC, PPC, LOVS, and TOLDI demonstrate robust feature matching capabilities. Conversely, SHOT and B-SHOT exhibit a marked decline in performance as noise levels escalate. While the effectiveness of SHOT and B-SHOT significantly diminishes with increased Gaussian noise, the remaining descriptors experience only marginal performance reductions, maintaining commendable feature matching capabilities. Among these, PPC ranks second, slightly trailing behind SGC. Additionally, as illustrated in Figure 7e–f, PPC continues to exhibit superior feature matching performance on both the UWAOR and Kinect datasets under conditions of added noise. Collectively, these findings underscore PPC’s substantial robustness in the face of noise interference, highlighting its potential applicability in diverse 3D computer vision tasks under varying environmental conditions.

### 4.3. Robustness Test for Point Density Variation

**Figure 8:**
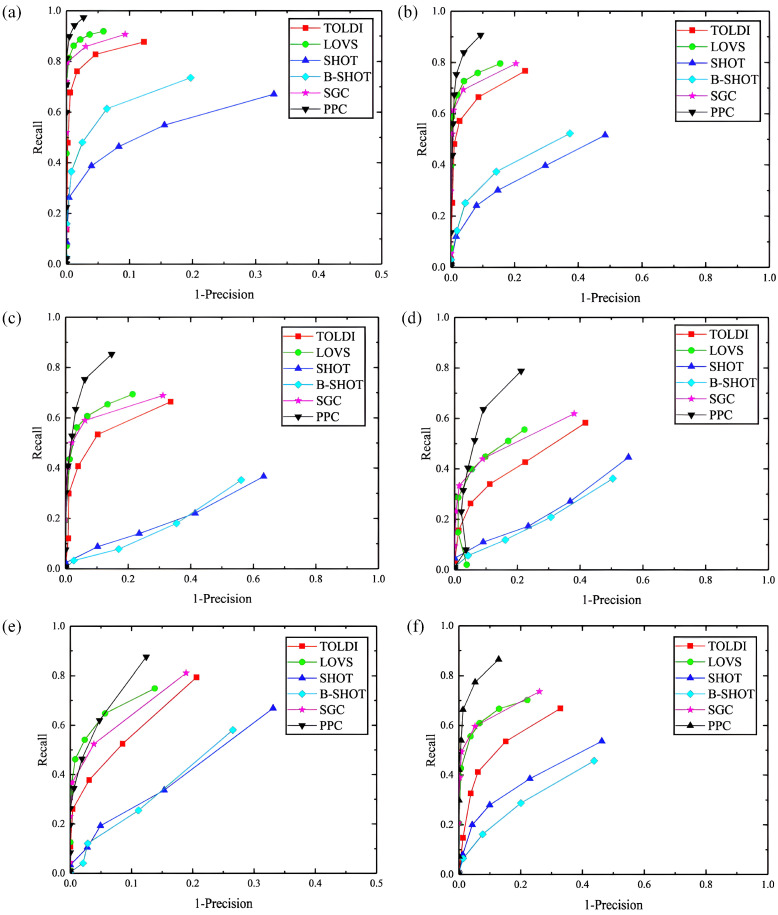
Robustness testing against point density variations. ((**a**), B3R dataset with 1/2 mesh decimation. (**b**), B3R dataset with 1/4 mesh decimation. (**c**), B3R dataset with 1/8 mesh decimation. (**d**), UWA dataset with 1/4 mesh decimation. (**e**), Kinect dataset with 1/4 mesh decimation. (**f**), B3R dataset with 0.3 mr Gaussian noise and 1/4 mesh decimation).

The robustness of the Planar Projection Contour (PPC) descriptor against variations in point density was evaluated through a series of tests. These tests involved assessing the feature matching performance of the scene point cloud under different downsampling levels (1/2, 1/4, and 1/8) using the B3R dataset, and the results were compared against those of established descriptors such as SGC, LOVS, SHOT, B-SHOT, and TOLDI. The experimentally generated Recall vs. 1 − Precision (RP) curves are displayed in Figure 8a–c. Further analysis was conducted on the UWAOR and Kinect datasets with a 1/4 downsampling rate, and the corresponding RP curves are depicted in Figure 8d–e. A comprehensive test to gauge PPC’s resilience to both Gaussian noise and point density variations was also performed by applying 1/4 downsampling to the scene point cloud from the B3R dataset, followed by the addition of 0.3 pr Gaussian noise. The RP curves from this experiment are illustrated in Figure 8f.

Observations from Figure 8a–c indicate that PPC consistently outperforms other descriptors across various downsampling levels on the B3R dataset, closely followed by LOVS and SGC. TOLDI’s performance was found to be slightly inferior to that of SGC, while SHOT and B-SHOT exhibited greater performance fluctuations. Additionally, PPC maintained the highest feature matching performance on the UWAOR and Kinect datasets, as evident from Figure 8d–e, suggesting that the convex hull contour’s feature representation offers considerable advantages in handling point density variations. Furthermore, Figure 8f demonstrates that PPC’s performance even remains superior under the combined influence of Gaussian noise and point density variations, affirming its robustness against these common challenges.

In summary, PPC’s architecture, centered around the convex hull contour feature representation, provides exceptional resilience to point density variations, establishing its robustness and effectiveness for various 3D computer vision applications.

### 4.4. PPC Calculation Efficiency Test

**Figure 9:**
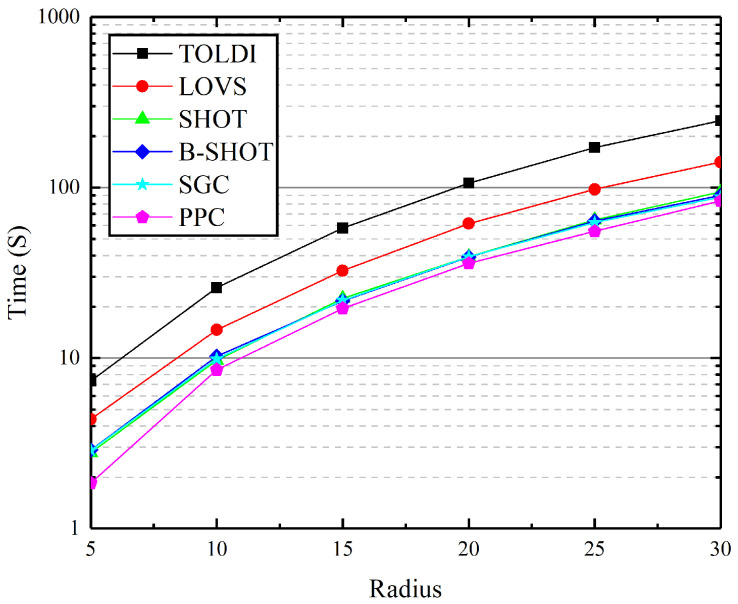
Descriptors’ computational efficiency tests.

To assess the computational efficiency of the Planar Projection Contour (PPC) descriptor, it was benchmarked against other established descriptors, including SGC [18], LOVS [19], SHOT [13], B-SHOT [16], and TOLDI [22]. This evaluation focused on measuring the total time required for representing the local features of 1000 key points across varying descriptor radius parameters, with radii including 10 mr, 15 mr, 20 mr, 25 mr, and 30 mr. The efficiency of each descriptor was quantified based on the total time consumed for feature representation, with the findings illustrated in Figure 9.

The analysis revealed that all descriptors exhibit high computational speed at smaller radii. However, as the radius *r* increases, the complexity involved in establishing the Local Reference Frame (LRF) and the extended dimensions of the descriptor result in a notable increase in computation time for TOLDI. Similarly, LOVS experiences a significant surge in computation time. In contrast, the increases in computation time for SGC, SHOT, and B-SHOT are more gradual. When compared to SGC, SHOT, and B-SHOT, PPC demonstrates superior performance, showcasing its enhanced computational efficiency.

This comparison highlights PPC’s advantage in terms of computational efficiency, especially as the complexity of the descriptor’s calculation increases with larger radii. PPC’s ability to maintain a lower computation time while ensuring accurate and robust feature representation makes it an attractive option for applications requiring efficient processing of point cloud data.

### 4.5. Application to 3D Matching

**Figure 10:**
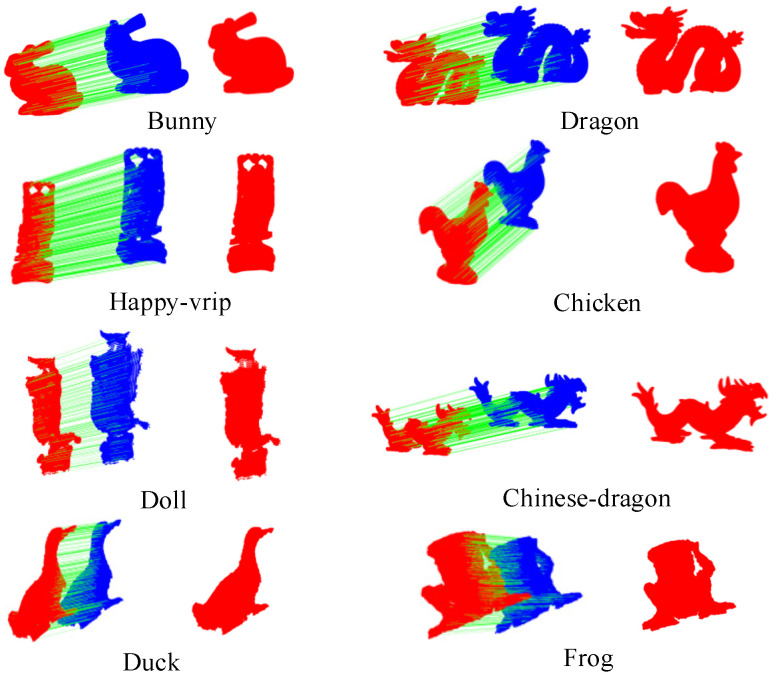
Example of 3D matching results. (The model point cloud and the scene point cloud are represented by red and blue respectively, and the green line represents the feature matching relationship between them).

The effectiveness of the Planar Projection Contour (PPC) descriptor was further validated through 3D matching tests using the dataset mentioned earlier. These tests adhered to the matching process outlined in [23], with a notable distinction: the point cloud description task was conducted using the PPC descriptor, and the method for comparing descriptor similarity was the one proposed in this study. The dataset used for testing included a diverse range of point cloud types, spanning from complete to localized formations and varying from high to low quality. The outcomes of these matching tests are visually documented in Figure 10.

Analysis of the experimental results reveals that the point cloud matching techniques employing the PPC descriptor successfully matched pairs of point cloud slices across all tested scenarios. Specifically, the PPC descriptor demonstrated its ability to form robust feature matching connections, leading to superior matching results in point clouds of both low and high quality. This performance underscores the PPC’s highly descriptive nature, highlighting its effectiveness in accurately capturing and representing the nuanced geometric information present in point clouds. Such attributes make the PPC an invaluable tool for 3D matching tasks, especially in applications where precision and reliability are paramount.

## 5. Conclusions

In this study, we introduce the Planar Projection Contour (PPC) descriptor, a novel local feature descriptor for point clouds. This descriptor enhances the robustness and descriptiveness of feature matching by leveraging Local Reference Frames (LRFs), convex hull contour extraction, and a unique method for assessing the matching degree of PPC descriptors. The process encompasses three primary steps:

(1) For a given key point, its nearest neighbors are identified. The Z-axis is derived through a weighted covariance analysis based on the spatial relationship between these neighboring points and the key point. The X-axis is then determined by the sum of weighted projections of the neighborhood points onto a plane, leading to the construction of the LRF.
(2) The neighborhood points are projected onto three orthogonal planes defined by the LRF, representing the local surface interaction between the key point and its neighbors. These 2D projection points are then modeled into convex hull contours, which succinctly capture the essential geometric characteristics of the local point cloud structure.
(3) The feature matching process involves extracting the convex hull contours from the three orthogonal planes and computing the overlapping areas of corresponding PPC contours. The matching degree is determined by accumulating these areas, with the highest accumulation signifying the optimal feature match between PPC descriptors.

This methodology demonstrates the PPC descriptor’s ability to effectively match point cloud features across varying conditions, such as noise interference and point density variations. The PPC descriptor’s design, focusing on the geometric fidelity of the point cloud data and its computational efficiency, showcases its potential as a powerful tool for applications in 3D computer vision and point cloud analysis.

## Figures and Tables

**Table 1:** Descriptor specific parameters.

Descriptor	Radius	Parameters	Dimension	Type
SHOT	20 mr	32 × 11	352	Float
SGC	20 mr	8 × 8 × 8 × 2	1024	Float
LOVS	20 mr	9 × 9 × 9	729	Binary
B-SHOT	20 mr	32 × 11	352	Binary
TOLDI	20 mr	20 × 20 × 3	1200	Float
PPC	20 mr	1 × 3	3	Convex

## Data Availability

The original data presented in the study are openly available in https://graphics.stanford.edu/data/3Dscanrep/ (accessed on 27 March 2024).
